# Clinical effectiveness of HPV vaccine by age at vaccination: a matched case-control study

**DOI:** 10.1016/j.lana.2025.101225

**Published:** 2025-09-16

**Authors:** Carlos R. Oliveira, Eugene D. Shapiro, Sangini S. Sheth, Mallory K. Ellingson, Nicholaus P. Johnson, Erin L. Sullivan, Troy D. Querec, Elizabeth R. Unger, Linda M. Niccolai

**Affiliations:** a Department of Pediatrics, Section of Infectious Diseases and Global Health, Yale School of Medicine, New Haven, CT, USA; b Department of Biostatistics, Section of Health Informatics, Yale School of Public Health, New Haven, CT, USA; c Department of Biomedical Informatics and Data Science, Yale School of Medicine, New Haven, CT, USA; d Department of Epidemiology of Microbial Diseases, Yale School of Public Health, New Haven, CT, USA; e Department of Obstetrics, Gynecology, & Reproductive Sciences, Yale School of Medicine, New Haven, CT, USA; f Division of High-Consequence Pathogens and Pathology, National Center for Emerging and Zoonotic Infectious Diseases, Centers for Disease Control and Prevention, Atlanta, GA, USA

**Keywords:** HPV vaccination, Vaccine effectiveness, High-grade cervical lesions, Case-control study

## Abstract

**Background:**

Important questions remain about the extent to which human papillomavirus (HPV) vaccines are realizing their full potential in real-world settings. This study aimed to assess how age at the time of vaccination influences its effectiveness against HPV-attributable high-grade cervical lesions (HGCL).

**Methods:**

In this matched case-control study conducted in New Haven County, Connecticut, cases were vaccine-eligible women diagnosed with HGCL associated with HPV 16 or 18 from 1/1/2010 to 12/31/2019. Controls were women with normal Pap smear results, matched to cases by age, medical practice, and date of Pap test. Participants were interviewed and records were reviewed to ascertain vaccination history and possible confounders including sexual behaviors. Vaccine effectiveness (VE) by age at the time of vaccination was assessed using matched odds ratios (OR) and 95% confidence intervals (CI) derived from multivariable conditional logistic regression models. VE was calculated as (1 − OR) × 100%.

**Findings:**

A total of 524 women (132 cases and 392 controls) were included. The adjusted VE of >1 dose of HPV vaccine was 54% (95% CI: 8–77%, p = 0·03). When the first dose was given at ≤18 years of age VE was 75% (95% CI: 13–93%, p = 0·03), and when vaccinated >18 years VE was 43% (95% CI: −22 to 74%, p = 0·15).

**Interpretation:**

These data demonstrate that the full benefit of HPV vaccines may not be realized when administered at older ages. Continued and strengthened efforts should be made to ensure that recommendations for HPV vaccination of younger adolescents are followed.

**Funding:**

10.13039/100000002National Institutes of Health, 10.13039/100000048American Cancer Society, 10.13039/100000938Robert E. Leet and Clara Guthrie Patterson Trust, and 10.13039/100000030Centers for Disease Control and Prevention.

**Research in context**

**Evidence before this study**

Real-world estimates of vaccine effectiveness (VE) are essential for monitoring its impact in clinical and public health practice. We searched Medline and EMBASE for studies published between 2007 and 2023 that quantified the effectiveness of human papillomavirus (HPV) vaccines by age at vaccination. Twenty-one studies from North America and Europe were identified, evaluating VE by age against outcomes such as HPV infection, anogenital warts, cervical abnormalities, and cervical cancer. Among these, ten studies specifically examined effectiveness against precancerous cervical abnormalities. Most studies employed retrospective cohort designs, utilizing data from national health registries, regional immunization or screening registries, and insurance claims databases. A consistent trend of declining vaccine effectiveness with increasing age at initiation was observed. Though these studies have made important contributions to the field, they have had several important limitations. Two recent systematic reviews found that almost all HPV VE studies had a serious risk of bias or confounding. Common biases included information bias in measuring the intervention or outcome. For example, most studies evaluated cytological or histological outcomes irrespective of HPV type. Further, nearly all of these previous studies used de-identified databases or population registries and thus were limited in their ability to assess and control for important confounders such as the number of sexual partners or age at sexual debut.

**Added value of this study**

This study builds on previous research by evaluating the effectiveness of the HPV vaccine based on the age of initiation in a socioeconomically diverse United States population. It addresses limitations of earlier studies by using HPV type-specific cases and disease-free, individually matched controls. Additional distinguishing features of this study include the comprehensive consideration of potential confounders through the collection of individual-level data via personalized surveys and a rigorous analytic approach aimed at minimizing residual confounding.

**Implications of all the available evidence**

The cumulative evidence, reinforced by the findings of this study, confirms the real-world benefits of initiating HPV vaccination at younger ages. These data can support healthcare providers in making strong recommendations for vaccinating at the recommended ages and help build public trust in the vaccination program.

## Introduction

Human papillomavirus (HPV) is a common infection and a cause of nearly all cases of cervical cancers.^1^ HPV vaccines have been available in the United States (US) since 2006 and are considered a cornerstone of cervical cancer prevention. Since the vaccine is preventive and does not have known therapeutic effects, vaccinating young adolescents is crucial to ensure they are protected before potential exposure to the virus. Presently, three HPV vaccines are authorized for use in the US. The 4-valent vaccine accounted for nearly all of the doses distributed in the US until it was replaced by the 9-valent vaccine in late 2016. The Advisory Committee on Immunization Practices (ACIP) recommends routine administration of HPV vaccine between ages 11 and 12 and encourages vaccination at the first opportunity, with the option to give it as early as 9 years of age.^2^ Despite the relatively high burden of HPV-related diseases,^3^^,^^4^ the uptake of HPV vaccine has consistently lagged behind other adolescent vaccines. A national survey in 2022 reported that by age 13, more than 85% of adolescents had received the tetanus, diphtheria, and acellular pertussis (Tdap) vaccine and the meningococcal conjugate vaccine (serogroups A, C, W, and Y), whereas only half were up to date with the HPV vaccine.^5^

Vaccine hesitancy is a multifaceted challenge. A large body of evidence indicates that this hesitancy among parents is primarily rooted in concerns over the safety, efficacy, necessity, and duration of protection.^6^ The initial licensure of the HPV vaccines was based on large-scale clinical trials that demonstrated that the vaccines were safe and efficacious (>95%) at preventing vaccine-type precancerous cervical lesions in healthy young adult women (mean age of 20 years).^7^ However, the recommendation for routine vaccination at age 11 or 12 was informed by immunobridging (not efficacy) studies. In these studies, efficacy was inferred by demonstrating that adolescents generated antibody titers following vaccination that were non-inferior to those observed in the adult trials.^8^ While immunobridging is sufficient for licensure, it does not provide direct evidence of benefit.

Several post-licensure studies have underscored the importance of vaccinating at younger ages.^9^, ^10^, ^11^ However, these studies have been limited by methodological issues. For instance, most relied solely on pre-existing data from medical records or anonymized registries, which often do not contain key confounding information such as sexual behavior. Thus, there is still a need for a rigorous assessment of vaccine effectiveness (VE) in adolescents as it is being used in real-world clinical practice settings.

Vaccine hesitancy also affects healthcare providers.^6^ Several studies have shown that the perception that there is not a pressing need to administer the vaccine contributes significantly to weaker recommendations, resulting in low uptake.^12^ According to a recent nationwide survey, nearly a third of pediatricians postponed vaccine initiation based on their judgment of how likely an adolescent was to engage in sexual activities.^13^ Furthermore, healthcare providers are also influenced by their perception of parents' hesitancy in a way that influences their recommendations.^6^ Real-world data demonstrating the greater benefits of vaccinating at recommended ages can support healthcare providers in providing strong recommendations and in building public trust in the vaccination program.^14^ To address this need, this study aimed to estimate the extent to which age at the time of vaccination influences VE against vaccine-type precancerous cervical lesions with robust control for potential confounders. Controlling for potential confounding is critical for valid estimates due to differences in demographic characteristics and sexual histories between women who are or are not vaccinated against HPV in actual practice.

## Methods

### Study design

Vaccine effectiveness was estimated using a matched case-control study design. For this study, the sampling frame was restricted to female residents of New Haven County, Connecticut who were born in or after 1981, had undergone cervical cancer screening, and received care within the Yale New Haven Health System (YNHHS) between 1/1/2010 and 12/31/2019. The age restriction was included to ensure participants were eligible for HPV vaccination, i.e., age 26 years or younger in 2006, the year the first vaccine received initial approval for this age group. YNHHS is the largest healthcare provider in Connecticut, with more than 2 million outpatient encounters annually. Clinical sites within this system are dispersed throughout the state but are heavily concentrated in New Haven County (population 855,000 individuals). The YNHHS criterion was included to facilitate the identification of controls who were matched on medical practice to control for confounding and to ensure comparable access to the medical records of both cases and controls.

#### Case selection

Cases were women with a biopsy-confirmed high-grade cervical lesion (HGCL) attributed to HPV 16 or 18. Histologic findings consistent with cervical intraepithelial neoplasia (CIN) grades 2, 2/3, 3, or adenocarcinoma in situ (AIS) were classified as HGCL. All three of the approved HPV vaccines prevent HPV 16/18 infections, which are the genotypes responsible for more than 70% of cervical cancers.^15^ Cases were identified in collaboration with the Connecticut Department of Public Health (CT DPH) and the Centers for Disease Control and Prevention (CDC)-funded population-based surveillance system known as HPV Impact Monitoring Project Across Connecticut (HPV-IMPACT).^16^ The surveillance conducted by CT DPH and HPV-IMPACT included the systematic collection of case reports from all pathology laboratories that serve residents of Connecticut. Enhanced surveillance was conducted for residents of New Haven County, whereby residual cervical tissue specimens from individuals with HGCLs were collected from clinical pathology laboratories and transferred to the CDC for genotyping, as previously described.^16^, ^17^, ^18^ Our analysis was restricted to patients with HGCL attributable to HPV types 16 or 18. HPV attribution was determined through detection and typing tests conducted by the CDC directly on cervical biopsy samples with HGCL. If cervical biopsy specimens were unavailable or the genotyping results were inconclusive, HPV attribution was determined using assays performed as part of routine clinical care at Yale Pathology on the cervical cytology specimen that led to the biopsy. The HPV detection and typing methods are detailed in the Supplemental Methods.

#### Control selection

Controls were women with normal Pap smear results and were individually matched to cases by age, medical practice, and date of Pap test. Normal Pap smear results were defined as specimens reported both as “satisfactory for evaluation” and “negative for intraepithelial lesion or malignancy”. Matched controls were identified using a previously validated natural language processing (NLP) algorithm.^19^ This algorithm searched all cervical Pap smears performed at Yale Pathology and generated a list of potential matched controls for a given case based on the three matching criteria: age (±12 months), date of pap smear (±12 months), and practice (within the YNHHS).

### Recruitment strategy

The enrollment period lasted from 10/1/2016 to 9/30/2021. All eligible patients who were diagnosed with HGCL and tested positive for HPV 16 and/or 18 between 1/1/2010 and 12/31/2019 were invited to participate. Following enrollment of each case, the matching algorithm generated a list of potential controls. If more than two potential matched controls were identified, the controls were ordered based on how closely they matched the case by age and date of Pap smear. Letters of invitation outlining the study objectives and offering an option to opt out were mailed to the ten closest set of matched controls. After a two-week interval to allow for opt-outs, research staff initiated telephone contact with those who had not declined participation. If fewer than two controls per case were enrolled, and all invitees in the initial set had been reached or called at least five times, the process was repeated with the next set of ten matched controls. Invitations were not withdrawn once extended. Thus, controls who received an invitation letter and later expressed interest in participating were permitted to enroll even if we had already enrolled two controls for their respective case. This resulted in an average of three controls per case.

### Data collection

Standardized interviews and questionnaires were used to collect data on prior sources of medical care and potential confounding factors, including social determinants of health and behavioral histories. A detailed account of the self-reported sociodemographic factors (race/ethnicity, education, zip code of residence, type of health insurance, and socio-economic status) and behavioral factors (sexual activity and history, use of contraception, sexually transmitted infection (STI) history, smoking history, healthcare access and utilization) that were ascertained can be found in Table S1. Self-reported data were collected using a previously validated computer-assisted self-interviewing instrument.^20^ Participants who did not have access to a personal computer or a smartphone were given the option of completing the survey on a study tablet during an in-person visit or on paper by mail. Survey items were individualized for each participant so that all the relevant questions referred to the period prior to focal time. Focal time was defined for cases as the date of the Pap smear that immediately preceded the cervical biopsy in which the HGCL was identified (i.e., “trigger Pap”). For controls, focal time was defined as the date of the normal Pap smear that was individually matched to an enrolled case.

### Exposure history

To determine HPV vaccination status, we reviewed medical records from all prior sources of care identified by the participants, using identical methods for both cases and matched controls. For YNHHS-affiliated practices, trained staff extracted vaccination histories directly from the integrated electronic health record. For non-YNHHS practices, research staff conducted on-site medical record reviews or requested that providers complete standardized vaccination information forms. Only HPV vaccine doses documented in the medical record or verified by provider report, with associated administration dates, were included in the analyses.

### Statistical analyses

For the primary analysis comparing cases and matched controls, we assessed covariate balance using matched standardized mean differences (SMDs) and calculated p values using conditional logistic regression. Matched SMDs were defined as the mean within-set difference between each case and the average of its controls, divided by the standard deviation of these differences across sets. We also performed descriptive analyses to assess potential selection bias by comparing enrolled participants with those invited who declined participation. For this unmatched comparison, we calculated unmatched SMDs and evaluated statistical significance using Wilcoxon rank-sum and χ^2^ tests. Categorical variables with low counts were collapsed as detailed in Supplemental Methods. VE was estimated using the matched-odds ratio (OR) from conditional logistic regression models, using vaccination status as the exposure variable and case/control status as the outcome so that VE = 1 − OR × 100%. For our primary analysis, vaccine doses were defined *a priori* to be valid only if received ≥2 years before focal time to account for the latency between incident HPV infections and the development of HGCL. To test this assumption, sensitivity analyses were conducted that examined the effect of using different time intervals, specifically vaccination ≥6 months, ≥1 year, and ≥3 years prior to the focal time.

Multivariable conditional logistic regression was used to calculate VE stratified by age at the time of first vaccination while adjusting for the effects of possible confounders. The fully adjusted model retained the covariates that either changed the crude OR by ≥10% when included in the model or were statistically significant (p < 0·05) in the bivariate analysis. The results are presented as odds ratios (OR) and adjusted ORs (aOR) with 95% confidence intervals (CI). Formal statistical testing for effect modification was performed by including an interaction term in the conditional logistic regression framework and then comparing the coefficients for the different vaccinated age groups.

Sensitivity analyses were conducted to test for residual selection bias and to verify the stability of the overall estimates of VE. First, to examine possible heterogeneity in VE according to the outcome, VE was stratified by the histologic grade of the lesion (CIN 2 and CIN 2/3 vs. CIN3+ and AIS) and by the number of high-risk HPV types identified in the specimen. Finally, we used a Bayesian model averaging (BMA) framework to systematically evaluate alternative modeling specifications, test the robustness of our results, and to generate model-averaged estimates of VE that incorporate modeling uncertainty into the parameter estimation.^21^ For all analyses, a type I error of 5% (two-sided) was used to test for statistical significance. With 132 case-control sets and a 2:1 control-to-case ratio, this study had ≥80% power to detect a VE of at least 50% (two-tailed alpha < 0·05). All analyses were conducted in R, version 4.3.1 and STATA version 17.0. Further details on study definitions, statistical analysis, and sample size derivation are provided in the Supplemental Methods.

### Ethics approval

This study was approved on April 21, 2015 by the Yale University Institutional Review Board (HIC: 1502015308). It was also approved by the Connecticut DPH Human Investigation Committee (Protocol #818). All study participants provided written informed consent.

### Role of the funding source

The CDC participated in the parent study's design, laboratory testing, interpretation, manuscript preparation, and submission. The other funders had no role in study design, data collection, data analysis, data interpretation, or writing of the report.

## Results

### Study population

A total of 517 eligible patients with HGCL associated with HPV 16/18 were identified during the enrollment period. Up to five attempts were made to reach each eligible patient using a combination of physical mail, email, and telephone calls. In total, 277 (54%) of the eligible cases were reached and 132 were enrolled in the study (Figure S1). Overall, 84% (111/132) of the enrolled cases had HPV detection and typing performed at the CDC; the remaining were typed at Yale Pathology as part of routine clinical care. HPV 16 was detected in 95% of the cases (125/132) and HPV 18 was detected in 7% of the cases (9/132), of which 2 were co-infections with HPV 16. The cervical pathology was classified as CIN2 or CIN2/3 in 55% of the cases (73/132) and as CIN3 or AIS in 45% (59/132).

A total of 2351 potential controls were matched to the enrolled cases, of whom 796 (34%) were contacted during the study period and 392 were enrolled. Enrolled patients did not differ significantly from those not enrolled with respect to race/ethnicity, age, or socioeconomic status (Table S2).

The characteristics of cases and controls included in the study are shown in Table 1. The median age of the case patients was 28 years (interquartile range [IQR], 25–31). Cases were significantly more likely than their matched controls to have been uninsured (9·4% vs. 2·9%, p = 0·0029), to have smoked daily (22·5% vs. 8·5%, p < 0·0001), to have had their first sexual encounter before 15 years of age (20·0% vs. 11·7%, p = 0·0074), to have had four or more sexual partners prior to focal time (64·8% vs. 47·5%, p = 0·0003), and to have had a history of other sexually transmitted infections (29·1% vs. 18·1%, p = 0·016).

**Table 1:** Characteristics of cases and matched controls, N = 524.

Characteristic	Cases (N = 132)	Controls (N = 392)	SMD	p-value
**Age, median (IQR), year**	27·0 (24·0–30·0)	28·0 (25·0–31·0)	−0·10	0·50
**Race or ethnic group**				
Hispanic or Latinx	26/132 (19·7%)	68/392 (17·3%)	0·06	0·88
White, non-Hispanic	81/132 (61·4%)	219/392 (55·9%)	0·10	Reference
Black, non-Hispanic	18/132 (13·6%)	68/392 (17·3%)	−0·09	0·26
Mixed or other race	7/132 (5·3%)	37/392 (9·4%)	−0·14	0·14
**Education**				
≤High school graduate	31/131 (23·7%)	65/389 (16·7%)	0·17	Reference
≥College	100/131 (76·3%)	324/389 (83·3%)	−0·17	0·06
**Health insurance**				
Private	83/128 (64·8%)	284/380 (74·7%)	−0·21	Reference
Public	33/128 (25·8%)	85/380 (22·4%)	0·09	0·15
Uninsured	12/128 (9·4%)	11/380 (2·9%)	0·18	0·0029
**Relationship status**				
Married or committed	52/131 (39·7%)	174/390 (44·6%)	−0·08	Reference
Not married or other	79/131 (60·3%)	216/390 (55·4%)	0·08	0·41
**Smoking status**				
Daily smoker	29/129 (22·5%)	33/388 (8·5%)	0·31	<0·0001
Occasional smoker	25/129 (19·4%)	62/388 (16·0%)	0·10	0·07
Non-smoker	75/129 (58·1%)	293/388 (75·5%)	−0·33	Reference
**Age of first intercourse**				
≥15 years	100/125 (80·0%)	324/367 (88·3%)	−0·22	Reference
<15 years	25/125 (20·0%)	43/367 (11·7%)	0·22	0·0074
**Lifetime sex partners**				
≤1 partner	14/122 (11·5%)	101/360 (28·1%)	−0·35	Reference
2–3 partners	29/122 (23·8%)	88/360 (24·4%)	−0·13	0·07
≥4 partners	79/122 (64·8%)	171/360 (47·5%)	0·41	0·0003
**Use of condoms**				
No	90/127 (70·9%)	261/370 (70·5%)	0·04	0·80
Yes	37/127 (29·1%)	109/370 (29·5%)	−0·04	Reference
**History of STIs**				
No	90/127 (70·9%)	299/365 (81·9%)	−0·21	Reference
Yes	37/127 (29·1%)	66/365 (18·1%)	0·21	0·016
**Prior pregnancy**				
No	65/128 (50·8%)	216/366 (59·0%)	−0·17	Reference
Yes	63/128 (49·2%)	150/366 (41·0%)	0·17	0·08
**Healthcare utilization**^a^				
Clinic visits, median (IQR)	22·0 (11·5–42·5)	25·5 (13·5–45·0)	−0·05	0·57

Data are presented as median and interquartile range (IQR) for continuous measures, and n/total (%) for categorical. Percentages may not total 100 because of rounding. p-values estimated using conditional logistic regression. Mixed or other race: American Indian or Alaska Native, Asian, Pacific Islander and Native Hawaiian, or more than one race. STIs = sexually transmitted infections.

a Data from medical record review from all reported sources of care, number of visits since 1/1/2006. SMD = Standardized Mean Difference. Calculated as the mean within-set difference between each case and the average of its matched controls, divided by the standard deviation of these differences across sets. Data are all self-reported from questionnaires unless otherwise noted, see Table S1 for more details.

HPV vaccination at least 2 years prior to focal time was documented for 17% of cases and for 27% of the matched controls. Among the 128 vaccinated subjects, most (80%) had documentation of three doses prior to focal time (Table 2). The median age at the time of the first dose was 21 years (IQR: 18–23). Controls were more likely to have been vaccinated if they were non-Hispanic white (69% vs. 51%, p = 0·0041), younger (median age 26 vs. 28, p < 0·0001), single (72% vs. 49%, p < 0·0001), used condoms (82% vs. 66%, p = 0·0031), and had previously been pregnant (70% vs. 55%, p = 0·012) (Table S3). No patient characteristics were associated with vaccination among the cases (Table S4).

**Table 2:** HPV vaccine doses received by case-control status.

Characteristic	Total, N = 524	Cases, N = 132	Controls, N = 392
**≥1 dose any time before focal time**			
No	383/524 (73·1%)	105/132 (79·5%)	278/392 (70·9%)
Yes	141/524 (26·9%)	27/132 (20·5%)	114/392 (29·1%)
**Time between 1st HPV dose and focal time**			
Median (IQR), months	57·0 (40·0–88·0)	49·0 (27·0–83·0)	57·5 (43·0–89·0)
**≥1 dose ≥2 years before focal time**			
No	396/524 (75·6%)	109/132 (82·6%)	287/392 (73·2%)
Yes	128/524 (24·4%)	23/132 (17·4%)	105/392 (26·8%)
**Total number of doses before focal time**			
1 dose	13/128 (10·2%)	0/23 (0·0%)	13/105 (12·4%)
2 doses	13/128 (10·2%)	3/23 (13·0%)	10/105 (9·5%)
≥3 doses	102/128 (79·7%)	20/23 (87·0%)	82/105 (78·1%)
**Age of first dose**			
≤18 years	34/128 (26·6%)	4/23 (17·4%)	30/105 (28·6%)
>18–22 years	55/128 (43·0%)	13/23 (56·5%)	42/105 (40·0%)
>22 years	39/128 (30·5%)	6/23 (26·1%)	33/105 (31·4%)
Median age (IQR), years	21·0 (18·0–23·0)	21·0 (19·0–23·0)	21·0 (18·0–23·0)

Data are presented as median interquartile range (IQR) for continuous measures, and n/total (%) for categorical measures.

### Estimates of VE

**Fig. 1:**
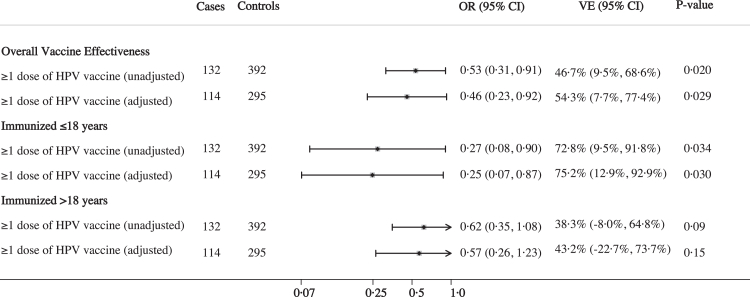
**Estimated effectiveness of HPV vaccine by age at the time of immunization.** Shown is the overall adjusted and unadjusted HPV vaccine effectiveness (VE) against high-grade cervical lesions that are attributable to HPV types 16 or 18. Adjusted models control for insurance, age of first intercourse, number of sex partners, smoking history, self-reported race/ethnicity, and history of other sexually transmitted infections. Bars indicate 95% confidence intervals. The reference category was unvaccinated at least 2 years before focal time.

The overall unadjusted VE of one or more doses of HPV vaccine when given at least two years prior to focal time was 47% (95% CI: 10–69%, p = 0·020) (Fig. 1). The final adjusted model included 6 variables: insurance, age of first intercourse, lifetime number of sex partners, smoking history, self-reported race, and history of STI (Table S5). Controlling for these potential confounders, the overall adjusted VE (aVE) was 54% (95% CI: 8–77%, p = 0·029). Age at the time of vaccination was the most influential covariate, followed by smoking history (Table S6). Stratifying by age at the time of vaccination revealed a 32-percentage-point difference in VE between age groups. The aVE was 75% (95% CI: 13–93%, p = 0·030) for those vaccinated at ≤18 years, compared to 43% (95% CI: −22 to 74%, p = 0·15) for those vaccinated at >18 years. While substantial, this difference did not reach statistical significance in a formal test for interaction (p = 0·24).

### Sensitivity analyses

**Fig. 2:**
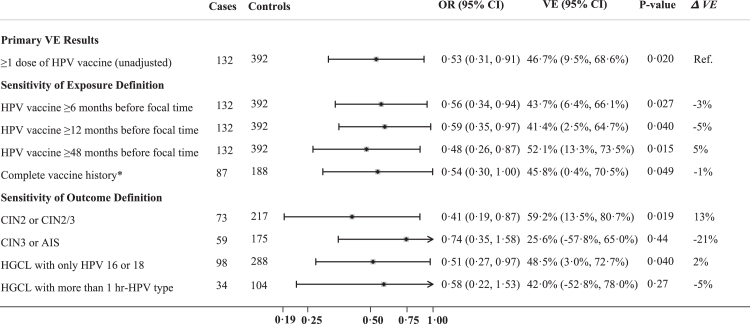
**Exposure definition, outcome definition, and other sensitivity analyses.** Shown is the overall HPV vaccine effectiveness (VE) against high-grade cervical lesions that are attributable to HPV types 16 or 18 and sensitivity analyses exploring possible heterogeneity in overall VE based on different exposure and outcome definitions. Bars indicate 95% confidence intervals. The reference category was unvaccinated at least 2 years before focal time. hr-HPV type = high-risk HPV type, which includes HPV 16, 18, 31, 33, 35, 39, 45, 51, 52, 56, 58, 59, 66, and 68. Δ VE is the difference between primary VE results and sensitivity analysis. ∗Excluding 42 cases and 16 controls who had 1 or more missing or unavailable vaccine records.

**Fig. 3:**
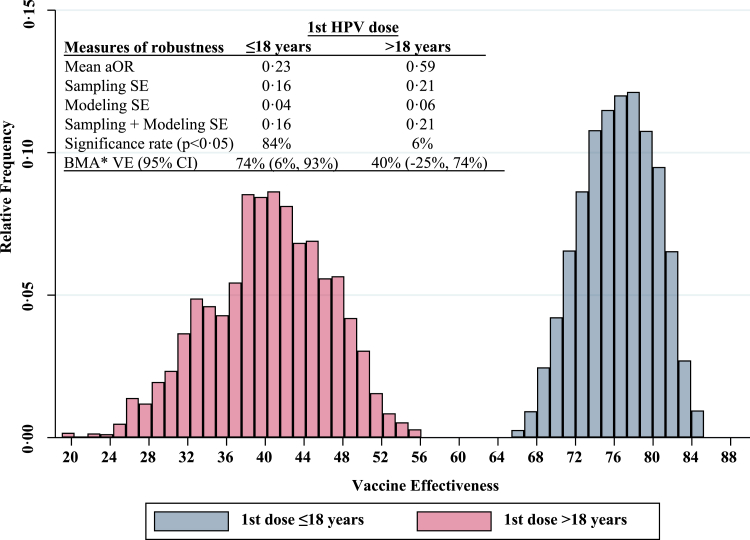
**Modeling distribution of vaccine effectiveness by age of immunization, N = 8192 models.** aOR = adjusted odds ratio; VE = vaccine effectiveness; SE = standard error; Significance rate = the percentage of models that report a statistically significant VE. ∗BMA = Bayesian model averaging. Only includes models that had a Posterior Model Probability ≥0·01. Relative Frequency refers to the proportion of models that support a specific VE estimate within the ensemble of models considered.

When shorter latency periods (<2 years) were specified, there was a non-significant 3–5% decrease in the estimated VE. The VE was 5% lower against HGCLs that had more than 1 high-risk type, though confidence intervals crossed the null (Fig. 2). Additional sensitivity analyses were conducted to address modeling uncertainty. Using a BMA framework, an initial set of 8192 distinct candidate models were fit and considered for the analyses of overall VE and for VE by age at the time of vaccination. Fig. 3 shows the modeling distribution across all models. The estimated VE for individuals vaccinated at ≤18 years of age was robust to the choice of potential confounders, maintaining statistical significance (p < 0·05) in 84% of all models considered. The components of the top subset of models, their model probability, and the posterior inclusion probability of each potential confounder are summarized in Figures S2 and S3. After systematically evaluating all candidate models, the model-averaged aVE varied from the primary results by <3%.

## Discussion

HPV vaccines were licensed based on pre-licensure placebo-controlled trials that found them to be safe and efficacious (>95%) at preventing HGCL associated with HPV types 16 and 18. In this real-world study evaluating HPV vaccine effectiveness within a diverse U.S. population, we found that one or more doses provided 54% (95% CI: 8–77%) protection against HGCL attributable to HPV types 16/18. The discrepancy between prelicensure efficacy and our observed effectiveness is likely driven by late vaccine initiation in our sample, where the median age at first vaccination was 21 years—well above the recommended 11–12 years. Because HPV vaccines are prophylactic, they do not protect against infection by HPV vaccine types to which individuals have been previously exposed that often occurs by early adulthood.^22^ The potential consequence of late vaccination is supported by our age-stratified results. When the first dose of the vaccine was administered at ≤18 years of age, effectiveness was strong and statistically significant at 75%, whereas effectiveness in those vaccinated after 19 years of age was lower at 43% and not statistically significant. While the difference between the two age groups did not meet the threshold for statistical significance (p = 0·24), the direction and magnitude of the effect is suggestive of reduced effectiveness at older ages.

This observed pattern of diminishing effectiveness with older age at vaccination is consistent with findings from other post-licensure studies. For instance, Hofstetter et al. found a 76% lower risk of cervical abnormalities among females who began the vaccination series between the ages of 11 and 14.^23^ This protective effect was noted to diminish for those who initiated vaccination past the age of 18 (estimated aVE = 15%). Another study, utilizing data from an integrated healthcare system in the US, estimated VE to be 39% (95% CI: 19–54%) for the first dose received between ages 14 and 17, 28% (95% CI: 10–42%) for those aged 18–20, and 6% (95% CI: −9 to 19%) for individuals aged 21 years or older.^24^

Despite the valuable insights these earlier studies provided, they had some limitations, including incomplete control for confounding. Because post-licensure VE studies typically do not use randomized designs, meticulous adjustment for potential confounders is essential. One of the distinguishing factors of this study is the implementation of measures to account for known confounders such as age, sexual behavior patterns, and smoking habits. This was accomplished by synthesizing data from multiple sources, including administrative records, clinician notes, and self-reported information. Among the potential confounders considered, smoking status and sexual risk behaviors—including age at first intercourse, lifetime number of sexual partners, and history of STIs—were identified as important factors. These variables function to some extent as proxies for behaviors that increase the risk of acquiring HPV infection. However, they also may play a more direct role in cervical carcinogenesis after HPV infection. Various components of tobacco smoke, for example, are known mutagens capable of independently promoting carcinogenesis in cervical epithelial cells.^25^ There is also a growing body of evidence showing how HPV co-infection with other STIs, such as chlamydia, can promote HPV persistence and progression to cervical intraepithelial neoplasia.^26^ While these factors lend explanatory depth to our analytical models, our central finding—the effectiveness by age at the time of immunization—proved to be robust even after adjusting for these factors. These data add to the current body of evidence by incorporating a level of rigor and control for confounding not achieved in other studies to date.

Recent systematic reviews have also highlighted that most prior HPV VE studies utilized a case definition that did not consider the type of HPV involved in the disease, specifically if it was a type targeted by the vaccine.^9^^,^^10^ This is a critical factor because bias towards underestimating the true VE tends to occur when the case definition has low specificity.^27^ Previous HPV-IMPACT analyses have estimated the overall type-specific VE in the US using an indirect cohort approach, in which the proportions of vaccinated patients with HGCL were compared among those associated with HPV vaccine-types and those associated with non-vaccine-types.^17^^,^^18^ Potential limitations of the indirect cohort design are that it can underestimate VE if the vaccines confer cross-protection against non-targeted types, or it can overestimate VE if there is type replacement. Our study builds on this previous work by providing estimates of VE stratified by age of vaccination using type-specific outcomes, HGCL-free controls, and individual matching by age, practice, and focal time. This approach minimizes misclassification of case-control status and is less susceptible to confounding by secular trends, such as changes in clinical practice or changes in the prevalence of HPV in the community. Furthermore, it ensures that cases and controls have had a similar amount of time to be vaccinated or to develop the disease, leading to better comparability.

The study has certain limitations. First, our sample size precluded additional analyses examining VE evaluations based on the number of doses or type of vaccine received and VE across different socioeconomic strata. Given the known health inequities associated with HPV-related diseases, studies that directly explore these disparities remain a critical need.^28^ Additionally, the observed difference in VE by age (75% vs. 43%), while notable, occurred within the context of wide and overlapping confidence intervals. However, the lack of statistical significance in these age-stratified analyses should be interpreted cautiously, as the study lacked statistical power to detect differences of this magnitude. Second, despite efforts to compile comprehensive vaccine records, participant recall issues and record disposal at some sites may have led to incomplete data. Third, although all HPV-positive specimens in this study were typed, assays used for formalin-fixed paraffin-embedded tissues differed from the clinical assay used on cytology. Fourth, as is the case with most case-control studies, there is the potential for selection bias or residual confounding. To mitigate this, we used computer algorithms to systematically search for and select individually matched controls. We also conducted extensive model validations, uncertainty analyses, and internal consistency checks to minimize the potential for residual confounding and enhance the credibility of our findings. Lastly, the study's timing restricted the evaluation of VE against additional HPV types targeted by the newer 9-valent vaccine, which is the vaccine that is currently in use in the US for the prevention of cervical cancer. Continued monitoring is needed to ascertain the VE against these important clinical outcomes.

In conclusion, this study adds to the growing body of evidence demonstrating that timely vaccination is essential to optimize the effectiveness of HPV vaccines. These results support ongoing public health efforts to promote early vaccination and provide clinicians with data to guide conversations with patients and families about the timing of immunization.

## Contributors

CRO, EDS, SSS, and LMN contributed to the study concept, design, and funding acquisition. NPJ, CRO, and MKE developed the statistical methods and performed the statistical analysis. ELS, LMN, and CRO were responsible for project administration and oversaw data collection and curation. CRO and LMN directly accessed and verified the underlying data reported in the study and drafted the original manuscript. TDQ and ERU were responsible for laboratory quality management and HPV detection and typing on formalin-fixed paraffin-embedded sections. All authors reviewed the manuscript, interpreted data, and approved the final version for publication.

## Data sharing statement

External researchers can make written requests to the corresponding author for sharing of completely de-identified and aggregate-level data. Data are available for researchers to allow replication of results provided all ethical and legal requirements are met. Requests will be assessed on a case-by-case basis in consultation with the lead and co-investigators. All data sharing will abide by rules and policies defined by the involved parties. Data-sharing mechanisms will ensure that the rights and privacy of individuals participating in research will be protected at all times. The study protocol and statistical code used are also available on request from the corresponding author.

## Declaration of interests

CRO has received grants from National Institutes of Health (NIH), American Cancer Society (ACS), the Robert E. Leet and Clara Guthrie Patterson Trust Foundation, and has leadership roles in the American Academy of Pediatrics-Section on Epidemiology, Public Health, and Evidence (SOEPHE), Eastern Society for Pediatric Research (ESPR), and Journal of the Pediatric Infectious Diseases Society (JPIDS). LMN has received grants from NIH, CDC, served as Scientific Advisor for Merck, GSK, and served on a data safety monitoring board for Moderna and GlaxoSmithKline (GSK). SSS has served as a consultant for Merck, received grants from National Institute of Allergy and Infectious Disease (NIAID), CDC, and National Cancer Institute (NCI), and has leadership role in the American Society for Colposcopy and Cervical Pathology (ASCCP). MKE and EDS have received grant support from the NIH. All other authors declare no conflicts of interest.
