# Supplementary material for: Clinical effectiveness of HPV vaccine by age at vaccination: a matched case-control study
Source: Lancet Reg Health Am. 2025 Sep 16;51:101225. doi: 10.1016/j.lana.2025.101225 (PMC12465035; doi:10.1016/j.lana.2025.101225)
Supplement: Supplemental Materials [file mmc1.docx]

**SUPPLEMENTAL APPENDIX**

This appendix has been provided by the authors to give readers additional information about their work.

**TABLE OF CONTENTS**

| **SECTION 1. SUPPLEMENTAL TABLES REFERENCED IN THE MAIN MANUSCRIPT** | 1 |
| --- | --- |
| List of Abbreviations | 2 |
| Table S1. Study definitions | 2 |
| Table S2. Standardized mean difference (SMD) of cases and controls by enrollment status. | 4 |
| Table S3. Correlates of vaccination among controls, N=392 | 5 |
| Table S4. Correlates of vaccination among cases, N=132 | 6 |
| Table S5. Crude and adjusted OR of HGCL by vaccination status, overall and by age of vaccination. | 7 |
| Table S6. Variable influence analysis | 8 |
| Table S7. Study Team Members | 9 |
| **SECTION 2. SUPPLEMENTAL FIGURES REFERENCED IN THE MAIN MANUSCRIPT** |  |
| Figure S1. Flow diagram for the selection of subjects | 10 |
| Figure S2. Composition of top subset of candidate models used for estimating overall vaccine effectiveness. | 11 |
| Figure S3. Overall HPV vaccine effectiveness: model-averaged and the top subset of candidate models. | 12 |
| **SECTION 3. SUPPLEMENTAL METHODS** |  |
| Statistical analysis | 13 |
| Sensitivity analysis | 14 |
| HPV detection and typing | 14 |
| **SECTION 4. Supplement References** | 15 |

**List of Abbreviations**

| AAP-SOEPHE | American Academy of Pediatrics - Section on Early Professionals and Health Equity |
| --- | --- |
| ACIP | Advisory Committee on Immunization Practices |
| ACS | American Cancer Society |
| AIS | Adenocarcinoma In Situ |
| aOR | Adjusted Odds Ratios |
| ASCCP | American Society for Colposcopy and Cervical Pathology |
| aVE | Adjusted Vaccine Effectiveness |
| BMA | Bayesian Model Averaging |
| CASI | Computer-Assisted Survey Interviewing |
| CDC | Centers for Disease Control and Prevention |
| CI | Confidence Intervals |
| CIN | Cervical Intraepithelial Neoplasia |
| CT DPH | Connecticut Department of Public Health |
| DNA | Deoxyribonucleic Acid |
| ESPR | Eastern Society for Pediatric Research |
| GED | General Educational Development |
| GSK | GlaxoSmithKline |
| HGCL | High-Grade Cervical Lesions |
| HPV | Human Papillomavirus |
| HPV-IMPACT | Human Papillomavirus Vaccine Impact Monitoring Project Across Connecticut |
| IQR | Interquartile Range |
| JPIDS | Journal of the Pediatric Infectious Diseases Society |
| MD | Doctor of Medicine |
| MenACWY | Meningococcal conjugate vaccine (serogroups A, C, W, and Y) |
| MPH | Master of Public Health |
| MS | Master of Science |
| NCI | National Cancer Institute |
| NIAID | National Institute of Allergy and Infectious Diseases |
| NIH | National Institutes of Health |
| NIEL | Negative for Intraepithelial Lesion or Malignancy |
| NLP | Natural Language Processing |
| OR | Odds Ratio |
| PA | Physician Assistant |
| PAP | Papanicolaou (Pap smear) |
| PCR | Polymerase Chain Reaction |
| PhD | Doctor of Philosophy |
| PIP | Posterior Inclusion Probability |
| RN | Registered Nurse |
| SES | Socioeconomic Status |
| SMD | Standardized Mean Differences |
| STI | Sexually Transmitted Infection |
| Tdap | Tetanus, Diphtheria, and acellular Pertussis |
| US | United States |
| VE | Vaccine Effectiveness |
| YNHHS | Yale New Haven Health System |

# SECTION 1. SUPPLEMENTAL TABLES REFERENCED IN The MAIN MANUSCRIPT

## Table S1. Study definitions

| **Variable** | **Definition** |
| --- | --- |
| **Case**  Source:  HPV-IMPACT and Medical records | - Female sex (assigned at birth), born on or after 1981, resident of New Haven County, and patient of the Yale New Haven Health System, diagnosed with a High-Grade Cervical Lesion (HGCL) that tested positive for HPV 16 or HPV 18. - High-Grade Cervical Lesion (HGCL) is a cervical histology report with a primary diagnosis of Cervical Intraepithelial Neoplasia (CIN) grades 2 or 3 or adenocarcinoma in situ |
| **Control**  Source: Medical records | - Female sex (assigned at birth), with a normal pap smear, matched to a case by age (±12 months), date of pap smear (±12 months), and practice. - Normal Pap smear: defined as cervical cytology that was deemed satisfactory for evaluation by a clinical pathologist and was negative for intraepithelial lesion or malignancy (NIEL). |
| **Focal time** Source: HPV-IMPACT, Medical records | - The common date between cases and controls. For controls, the focal time was the date of the normal pap smear. For cases, the focal time was the date of the abnormal pap that led to a cervical biopsy and subsequent diagnosis of HGCL. |
| **Exclusion criteria**  Source: Medical records, interviews, HPV-IMPACT | - Unwilling or unable to sign informed consent. - Patients who explicitly opted out of research in the electronic health record - Patients with incomplete contact information in the electronic health record - Cervical samples that were inadequate or unsatisfactory for evaluation, thereby precluding confirmation of case/control status. |
| **Vaccinated**  Source: Medical records | - Only doses of the vaccine documented in the medical record or verified by the provider with dates were counted. - A subject who received a vaccine dose ≥2 years prior to focal time. |
| **Race and ethnicity**  Source: Questionnaire | - Survey question for Ethnicity: Are you Hispanic/Latino? - Survey responses for Ethnicity: (1) Yes; (2) No; (3) I don't know; (4) I do not wish to answer. - Survey question for Race: What is your race? (Select one or more responses.) - Survey responses for Race: (1) White; (2) Black or African American; (3) Native Hawaiian or Other Pacific Islander; (4) American Indian or Alaska Native; (5) Asian; (6) Other; (7) I don't know; (8) I do not wish to answer. - Analysis: Combined race and ethnicity into 4 groups: (a) Hispanic or Latinx, (b) White, non-Hispanic, (c) Black, non-Hispanic, and (d) Mixed or other race groups. |
| **Education**  Source: Questionnaire | - Survey question: What was the highest grade or year of school you completed in [PAP YEAR]? - Survey responses: (1) Never attended school or only attended kindergarten; (2) Grades 1-8 (Some elementary); (3) Grades 9-11 (Some high school); (4) Grade 12 or GED (High school graduate); (5) College 1 year to 3 years (Some college or technical school); (6) College ≥4 years (College graduate); (7) I don’t know. - Analysis: Collapsed in 2 groups: (a) High school graduate or less (responses 1-4), and (b) Some college or greater (responses 5, 6). |
| **Relationship status**  Source: Questionnaire | - Survey question: Prior to your Pap smear in [PAP YEAR], what was your marital status? - Survey responses: (1) Married; (2) Divorced; (3) Widowed; (4) Separated; (5) Never Married; (6) A member of an unmarried couple; (7) I don’t know; (8) Other. - Analysis: Collapsed into 2 groups: (a) Married or committed (responses 1, 6), and (b) Not Married or other (responses 2, 3, 4, 5, 8). |
| **Health insurance**  Source: Questionnaire | - Survey question: Prior to your Pap smear in [YEAR], did you have any kind of health insurance? - Survey responses: (1) Continuously insured with private insurance; (2) Continuously insured with government plans; (3) continuously insured with military plans; (4) Had insurance (private or government or military) for part of the year. (5) Was uninsured for the entire year. (6) I don’t know if I had insurance. - Analysis: Collapsed in 3 groups: (a) Private (response 1), (b) Public (response 2, 3), and (c) Uninsured (4, 5). |
| **Smoking status**  Source: Questionnaire | - Survey question: What is your cigarette smoking behavior in [PAP YEAR]? - Survey responses: (1) Daily smoker (at least one cigarette per day, disregarding religious fasting); (2) Occasional smoker (less than one cigarette per day); (3) Ex-smoker of cigarettes; (4) Non-smoker of cigarettes; (5) I don't know - Analysis: Collapsed in 3 groups: (a) Daily smoker (response 1); (b) Occasional smoker (responses 2, 3), and (c) Non-smoker (response 4). |
| **Age of 1st intercourse**  Source: Questionnaire | - Survey question: How old were you when you had vaginal intercourse for the first time? - Survey responses: (1) I have never had vaginal intercourse; (2) 11 years old or younger; (3) 12 years old; (4) 13 years old; (5) 14 years old; (6) 15 years old; (7) 16 years old; (8) 17 years old or older; (9) I don't know. - Analysis: Collapsed into 2: (a) <15 years (responses 2, 3, 4, 5) , and (b) ≥15 years (6, 7, 8). |
| **Lifetime sex partners**  Source: Questionnaire | - Survey question: Prior to your Pap smear in [PAP YEAR], with how many women or men have you had sexual intercourse (anal, vaginal or oral sex)? - Survey responses: (1) 1 man or woman; (2) 2 men or women; (3) 3 men or women; (4) 4 men or women; (5) 5 men or women; (6) 6 or more men or women; (7) I don't know. - Analysis: Collapsed into 3 groups: (a) ≤1 partners (response 1); (b) 2-3 partners (responses 2-3); ≥4 partners (response 5, 6). |
| **Condom use**  Source: Questionnaire | - Survey question: When you had sexual intercourse in [PAP YEAR], did you or your partner use a condom? - Survey responses: (1) Every time; (2) Most of the time; (3) Rarely; (4) Never; (5) I did not have sexual intercourse in [YEAR]; (6) I don't know. - Analysis: Analysis: Collapsed in 2 groups: (a) Yes (response 1, 2, 3), and (b) No (response 4). |
| **History of STIs**  Source: Questionnaire | - Survey question: Prior to your Pap smear in [PAP YEAR], had you ever been diagnosed with one of these sexually transmitted diseases? (Check all that apply.) - Survey responses: (1) HIV/AIDS; (2) Gonorrhea; (3) Chlamydia; (4) Syphilis; (5) Herpes simplex; (6) Trichomonas; (7) None of these; (8) I don't know. - Analysis: Collapsed into: (a) Yes (response 1-6), (b) No (response 7). |
| **Prior pregnancy**  Source: Questionnaire | - Survey question: Were you ever pregnant prior to your Pap smear in [PAP YEAR]? - Survey responses: (1) No; (2) 1 time; (3) 2 times; (4) 3 times; (5) 4 or more times; (6) I do not know. - Analysis: Collapsed into (a) Yes (responses 2-5), (b) No (response 1). |
| **Healthcare utilization**  Source: Medical records. | - Number of outpatient clinician encounters as early as 1/1/2006. This was obtained by counting visits from the electronic medical records and requesting counts from providers from all reported sources of care. Patients with >100 encounters were coded as 100. |
| **Socioeconomic status**  Source: Medical records. | - The most recent zip code of eligible and invited subjects was used as a proxy for socioeconomic status. This was accomplished by linking the subject’s zip code to the Census data to determine if the subject resided in a zip code where either a high (≥25%), medium (>5% to <25%), or low (≤5%) proportion of residents have incomes below the poverty level. |

**Table S2. Standardized mean difference (SMD) of cases and controls by enrollment status**

| **Characteristic** | **Cases (N=517)** | | **SMD** | **P value** | **Controls (N=2,351)** | | **SMD** | **P value** |
| --- | --- | --- | --- | --- | --- | --- | --- | --- |
|  | **Enrolled (N=132)** | **Not Enrolled (N=385)** |  |  | **Enrolled (N=392)** | **Not Enrolled (N=1,959)** |  |  |
| **Age, median (IQR), year** | 32  (30-34) | 32  (29-34) | 0·09 | 0·39 | 33  (30-35) | 33  (31-35) | -0·14 | 0·29 |
| **Race or ethnic group** |  |  |  | 0·92 |  |  |  | 0·33 |
| White, non-Hispanic | 81/132 (61·4%) | 218/385 (56·6%) | 0·05 |  | 219/392 (55·9%) | 976/1,959 (49·8%) | 0·02 |  |
| Black, non-Hispanic | 18/132 (13·6%) | 48/385 (12·5%) | 0·02 |  | 68/392 (17·3%) | 306/1,959 (15·6%) | 0·00 |  |
| Hispanic or Latinx | 26/132 (19·7%) | 83/385 (21·6%) | -0·07 |  | 68/392 (17·3%) | 272/1,959 (13·9%) | 0·05 |  |
| Other race^b^ | 7/132 (5·3%) | 21/385 (5·5%) | -0·02 |  | 37/392 (9·4%) | 221/1,959 (11·3%) | -0·10 |  |
| Unknown | 0/132 (0·0%) | 15/385 (3·9%) | - |  | 0/392 (0·0%) | 184/1,959 (9·4%) | - |  |
| **Socioeconomic status** |  |  |  | 0·20 |  |  |  | 0·21 |
| ≥25% poverty | 11/132 (8·3%) | 33/385 (8·6%) | -0·02 |  | 16/392 (4·1%) | 82/1,959 (4·2%) | 0·00 |  |
| >5% to <25% poverty | 91/132 (68·9%) | 228/385 (59·2%) | 0·18 |  | 239/392 (61·0%) | 1,305/1,959 (66·6%) | -0·09 |  |
| ≤5% poverty | 29/132 (22·0%) | 112/385 (29·1%) | -0·18 |  | 113/392 (28·8%) | 499/1,959 (25·5%) | 0·09 |  |
| Missing | 1/132 (0·8%) | 12/385 (3·1%) | - |  | 24/392 (6·1%) | 73/1,959 (3·7%) | - |  |

All variables were determined from electronic health records or the HPV-IMPACT surveillance system.

Percentages may not total 100 because of rounding.

Abbreviations: SMD, standardized mean difference; HPV, human papillomavirus.

SMD is the difference in means between cases and controls in units of the pooled standard deviation. Covariates with SMD >0·2 were considered to have important imbalances.

Socioeconomic status is estimated using the most recent zip code (see Table S1: study definitions).

Not enrolled includes patients who were reached and invited but opted not to participate or did not complete all study procedures (i.e., sign an informed consent and complete the study questionnaire).

P-value estimated using Pearson’s chi-squared for categorical variables and Wilcoxon rank-sum for continuous.

^b^ Other race included Asian, American Indian or Alaska native, native Hawaiian, pacific islander, and mixed race.

**Table S3. Correlates of vaccination among controls, N=392**

|  | **≥1 dose of HPV vaccine^a^** | |  |
| --- | --- | --- | --- |
| **Characteristic** | **Yes, N=105** | **No, N=287** | **P-value** |
| **Age, years** | 26·0 (24·0-29·0) | 28·0 (25·0-31·0) | <0·0001 |
| **Race or ethnic group** |  |  | 0.0041 |
| Hispanic or Latinx | 9/105 (8·6%) | 59/287 (20·6%) |  |
| White, non-Hispanic | 72/105 (68·6%) | 147/287 (51·2%) |  |
| Black, non-Hispanic | 13/105 (12·4%) | 55/287 (19·2%) |  |
| Mixed or other race group | 11/105 (10·5%) | 26/287 (9·1%) |  |
| **Education** |  |  | 0·36 |
| ≤ High school graduate | 14/104 (13·5%) | 51/285 (17·9%) |  |
| ≥ College | 90/104 (86·5%) | 234/285 (82·1%) |  |
| **Health insurance** |  |  | 0·08 |
| Private | 82/101 (81·2%) | 202/279 (72·4%) |  |
| Public | 15/101 (14·9%) | 70/279 (25·1%) |  |
| Uninsured | 4/101 (4·0%) | 7/279 (2·5%) |  |
| **Relationship status** |  |  | <0·0001 |
| Married or committed | 29/104 (27·9%) | 145/286 (50·7%) |  |
| Not married or other | 75/104 (72·1%) | 141/286 (49·3%) |  |
| **Smoking status** |  |  | 0·09 |
| Daily smoker | 4/104 (3·8%) | 29/284 (10·2%) |  |
| Occasional smoker | 15/104 (14·4%) | 47/284 (16·5%) |  |
| Non-smoker | 85/104 (81·7%) | 208/284 (73·2%) |  |
| **Age of 1st intercourse** |  |  | 0·86 |
| <15 years | 86/98 (87·8%) | 238/269 (88·5%) |  |
| ≥ 15 years | 12/98 (12·2%) | 31/269 (11·5%) |  |
| **Lifetime sex partners** |  |  | 0·018 |
| ≤ 1 partner | 21/95 (22·1%) | 80/265 (30·2%) |  |
| 2 to 3 partners | 17/95 (17·9%) | 71/265 (26·8%) |  |
| ≥ 4 partners | 57/95 (60·0%) | 114/265 (43·0%) |  |
| **Use of condoms** |  |  | 0·0031 |
| Yes | 82/100 (82·0%) | 179/270 (66·3%) |  |
| No | 18/100 (18·0%) | 91/270 (33·7%) |  |
| **History of STIs** |  |  | 0·22 |
| Yes | 77/99 (77·8%) | 222/266 (83·5%) |  |
| No | 22/99 (22·2%) | 44/266 (16·5%) |  |
| **Prior pregnancy** |  |  | 0·012 |
| Yes | 69/99 (69·7%) | 147/267 (55·1%) |  |
| No | 30/99 (30·3%) | 120/267 (44·9%) |  |
| **Healthcare utilization** |  |  | 0·020 |
| Number of outpatient visits | 20·0 (11·0-38·0) | 27·0 (15·0-47·0) |  |

Abbreviations: HPV, human papillomavirus; IQR, interquartile range; STIs, sexually transmitted infections.

Data are presented as median (IQR) for continuous measures, and n/total (%) for categorical.

Percentages may not total 100 because of rounding.

P-values estimated using Fisher's exact, Pearson's chi-squared, and Kruskal-Wallis for dichotomous, categorical, and continuous variables, respectively.

^a^ Received one or more doses of HPV vaccine 2 years before focal time.

**Table S4. Correlates of vaccination among cases, N=132**

|  | **≥1 dose of HPV vaccine^a^** | |  |
| --- | --- | --- | --- |
| **Characteristic** | **Yes, N=23** | **No, N=109** | **P-value** |
| **Age, years** | 26·0 (23·0-29·0) | 28·0 (25·0-31·0) | 0·07 |
| **Race or ethnic group** |  |  |  |
| Hispanic or Latinx | 4/23 (17·4%) | 22/109 (20·2%) | 0·49 |
| White, non-Hispanic | 17/23 (73·9%) | 64/109 (58·7%) |  |
| Black, non-Hispanic | 1/23 (4·3%) | 17/109 (15·6%) |  |
| Mixed or other race group | 1/23 (4·3%) | 6/109 (5·5%) |  |
| **Education** |  |  | 0·28 |
| ≤ High school graduate | 3/23 (13·0%) | 28/108 (25·9%) |  |
| ≥ College | 20/23 (87·0%) | 80/108 (74·1%) |  |
| **Health insurance** |  |  | 0·16 |
| Private | 19/23 (82·6%) | 64/105 (61·0%) |  |
| Public | 3/23 (13·0%) | 30/105 (28·6%) |  |
| Uninsured | 1/23 (4·3%) | 11/105 (10·5%) |  |
| **Relationship status** |  |  | 0·81 |
| Married or committed | 10/23 (43·5%) | 42/108 (38·9%) |  |
| Not married or other | 13/23 (56·5%) | 66/108 (61·1%) |  |
| **Smoking status** |  |  | 0·57 |
| Daily smoker | 4/23 (17·4%) | 25/106 (23·6%) |  |
| Occasional smoker | 3/23 (13·0%) | 22/106 (20·8%) |  |
| Non-smoker | 16/23 (69·6%) | 59/106 (55·7%) |  |
| **Age of 1st intercourse** |  |  | 0·07 |
| <15 years | 21/22 (95·5%) | 79/103 (76·7%) |  |
| ≥ 15 years | 1/22 (4·5%) | 24/103 (23·3%) |  |
| **Lifetime sex partners** |  |  | 0·30 |
| ≤ 1 partner | 4/22 (18·2%) | 10/100 (10·0%) |  |
| 2 to 3 partners | 3/22 (13·6%) | 26/100 (26·0%) |  |
| ≥ 4 partners | 15/22 (68·2%) | 64/100 (64·0%) |  |
| **Use of condoms** |  |  | 0·80 |
| Yes | 17/23 (73·9%) | 73/104 (70·2%) |  |
| No | 6/23 (26·1%) | 31/104 (29·8%) |  |
| **History of STIs** |  |  | 0·99 |
| Yes | 16/23 (69·6%) | 74/104 (71·2%) |  |
| No | 7/23 (30·4%) | 30/104 (28·8%) |  |
| **Prior pregnancy** |  |  | 0·17 |
| Yes | 15/23 (65·2%) | 50/105 (47·6%) |  |
| No | 8/23 (34·8%) | 55/105 (52·4%) |  |
| **Healthcare utilization** |  |  | 0·78 |
| Number of outpatient visits | 27·0 (12·0-38·0) | 22·0 (11·0-51·0) |  |

Abbreviations: HPV, human papillomavirus; IQR, interquartile range; STIs, sexually transmitted infections.

Data are presented as median (IQR) for continuous measures and n/total (%) for categorical.

Percentages may not total 100 because of rounding.

P-values were estimated using Fisher's exact, Pearson's chi-squared, and Kruskal-Wallis for dichotomous, categorical, and continuous variables, respectively.

^a^ Received one or more doses of HPV vaccine 2 years before focal time.

**Table S5. Crude and adjusted OR of HGCL by vaccination status, overall and by age of vaccination**

|  | **Bivariate Models** | | | **Adjusted Model 1: Vaccinated at any age** | | | **Adjusted Model 2: Vaccinated ≤ or > 18 years** | | |
| --- | --- | --- | --- | --- | --- | --- | --- | --- | --- |
| **Variables** | **OR** | **95%CI** | **P-value** | **aOR** | **95%CI** | **P-value** | **aOR** | **95%CI** | **P-value** |
| **≥ 1 Dose HPV vaccine^a^** |  |  |  |  |  |  |  |  |  |
| Unvaccinated |  | Reference |  |  | Reference |  |  | Reference |  |
| Vaccinated | 0·53 | [0·31,0·91] | 0·020 | 0·46 | [0·23,0·92] | 0·029 | - | - | - |
| Vaccinated ≤ 18 years | 0·27 | [0·08,0·90] | 0·034 | - | - | - | 0·25 | [0·07,0·87] | 0·029 |
| Vaccinated > 18 years | 0·62 | [0·35,1·08] | 0·09 | - | - | - | 0·57 | [0·26,1·23] | 0·15 |
| **Age, years** | 0·88 | [0·61,1·27] | 0·50 | - | - | - | - | - | - |
| **Race or ethnic group** |  |  |  |  |  |  |  |  |  |
| Hispanic or Latinx | 1·04 | [0·59,1·84] | 0·88 | 1·8 | [0·79,4·08] | 0·16 | 1·8 | [0·79,4·09] | 0·16 |
| White, non-Hispanic |  | Reference |  |  | Reference |  |  | Reference |  |
| Black, non-Hispanic | 0·71 | [0·39,1·29] | 0·26 | 0·95 | [0·37,2·43] | 0·92 | 0·92 | [0·36,2·35] | 0·85 |
| Mixed or other race | 0·53 | [0·23,1·23] | 0·14 | 1·69 | [5·44,5·28] | 0·36 | 1·64 | [0·52,5·15] | 0·39 |
| **Education** |  |  |  |  |  |  |  |  |  |
| ≤ High school graduate |  | Reference |  | - | - | - | - | - | - |
| ≥ College | 0·59 | [0·35,1·02] | 0·06 | - | - | - | - | - | - |
| **Health insurance** |  |  |  |  |  |  |  |  |  |
| Private |  | Reference |  |  | Reference |  |  | Reference |  |
| Public | 1·5 | [0·86,2·62] | 0·15 | 0·87 | [0·36,1·93] | 0·74 | 0·91 | [0·41,2·02] | 0·82 |
| Uninsured | 3·82 | [1·58,9·22] | 0·0029 | 5·2 | [1·59,16·97] | 0·0063 | 5·53 | [1·66,18·38] | 0·0052 |
| **Relationship status** |  |  |  |  |  |  |  |  |  |
| Married or committed |  | Reference |  | - | - | - | - | - | - |
| Not married or other | 1·19 | [0·79,1·80] | 0·41 | - | - | - | - | - | - |
| **Smoking status** |  |  |  |  |  |  |  |  |  |
| Daily smoker |  | Reference |  |  | Reference |  |  | Reference |  |
| Occasional smoker | 0·48 | [0·23,0·97] | 0·041 | 0·76 | [0·30,1·89] | 0·55 | 0·77 | [0·31,1·91] | 0·57 |
| Non-smoker | 0·29 | [0·16,0·52] | <0·0001 | 0·51 | [0·23,1·14] | 0·10 | 0·53 | [0·24,1·17] | 0·11 |
| **Age of 1st intercourse** |  |  |  |  |  |  |  |  |  |
| <15 years | 2·23 | [1·24,4·00] | 0·0074 | 2·52 | [1·14,5·53] | 0·022 | 2·62 | [1·18,5·85] | 0·018 |
| ≥ 15 years |  | Reference |  |  | Reference |  |  | Reference |  |
| **Lifetime sex partners** |  |  |  |  |  |  |  |  |  |
| ≤ 1 partner |  | Reference |  |  | Reference |  |  | Reference |  |
| 2 to 3 partners | 1·96 | [0·95,4·01] | 0·07 | 1·83 | [0·75,4·41] | 0·18 | 1·76 | [0·73,4·25] | 0·21 |
| ≥ 4 partners | 3·24 | [1·72,6·09] | 0.0003 | 2·87 | [1·26,6·51] | 0·012 | 2·75 | [1·22,6·25] | 0·015 |
| **Use of condoms** |  |  |  |  |  |  |  |  |  |
| Yes |  | Reference |  | - | - | - | - | - | - |
| No | 1·06 | [0·67,1·68] | 0·80 | - | - | - | - | - | - |
| **History of STIs** |  |  |  |  |  |  |  |  |  |
| Yes | 1·78 | [1·11,2·85] | 0·016 | 2·15 | [1·18,3·91] | 0·012 | 2·13 | [1·17,3·88] | 0·013 |
| No |  | Reference |  |  | Reference |  |  | Reference |  |
| **Prior pregnancy** |  |  |  |  |  |  |  |  |  |
| Yes | 1·47 | [0·95,2·27] | 0·08 | - | - | - | - | - | - |
| No |  | Reference |  | - | - | - | - | - | - |
| **Healthcare utilization** |  |  |  |  |  |  |  |  |  |
| No· of outpatient visits | 0·99 | [0·99,1·01] | 0·57 | - | - | - | - | - | - |

Abbreviations: OR, odds ratio; aOR, adjusted odds ratio; CI, confidence interval; HPV, human papillomavirus; HGCL, high-grade cervical lesion; STIs, sexually transmitted infections; VE, vaccine effectiveness.

Matched OR was calculated using bivariate conditional logistic regression with case/control status as the outcome.

aOR was calculated using conditional logistic regression retaining the covariates that, when included, changed the crude VE by ≥10% or were statistically significant on bivariate models (see Figure S1).

^a^ Only considers doses that were given ≥2 years before focal time. **Table S6. Variable influence analysis**

| **Variables** | **Mean ΔOR with variable inclusion** | **Percent ΔOR from unadjusted model*** |
| --- | --- | --- |
| Vaccinated > 18 years | 0·10 | 19% |
| Enrollment age, years | 0·00 | -1% |
| Race or ethnic group | -0·05 | -10% |
| Education | 0·00 | 0% |
| Relationship status | 0·00 | 0% |
| Smoking status | 0·06 | 11% |
| Age of first intercourse | -0·02 | -4% |
| Health insurance | 0·02 | 4% |
| Lifetime sex partners | -0·04 | -7% |
| Frequency of condom use | -0·01 | -2% |
| History of STIs | -0·04 | -8% |
| Prior pregnancy | 0·01 | 2% |
| Healthcare utilization | -0·01 | -2% |

Mean ΔOR = Mean difference in the odds ratio (OR) associated with the vaccine coefficient if the control variable is included in the model. Based on 8,192 models. *Corresponds to the percentage change in the OR when a given control variable is included in the model relative to the unadjusted (base) OR estimate. STI= sexually transmitted infections.

**Table S7. Study Team Members**

| **HPV-VE Study Team** | |
| --- | --- |
| Carlos R. Oliveira, MD | Yale School of Medicine, New Haven, CT, USA |
| Eugene D. Shapiro, MD | Yale School of Medicine, New Haven, CT, USA |
| Sangini S. Sheth, MD | Yale School of Medicine, New Haven, CT, USA |
| Mallory K. Ellingson, PHD | Yale School of Public Health, New Haven, CT, USA |
| Nicholaus P. Johnson, MPH | Yale School of Public Health, New Haven, CT, USA |
| Erin L. Sullivan | Yale School of Public Health, New Haven, CT, USA |
| Troy D. Querec, PhD | Centers for Disease Control and Prevention, Atlanta, GA, USA. |
| Elizabeth R. Unger, PhD | Centers for Disease Control and Prevention, Atlanta, GA, USA. |
| Linda M. Niccolai, PhD | Yale School of Public Health, New Haven, CT, USA |
| **Yale’s HPV-VE Study Recruitment Team** | |
| Anette Ortiz, PA | Yale School of Medicine, New Haven, CT, USA |
| Ashlynn Torres, MD | Yale School of Medicine, New Haven, CT, USA |
| Lital Avni-Singer, MD | Yale School of Medicine, New Haven, CT, USA |
| Deborah Rardin, RN | Yale School of Medicine, New Haven, CT, USA |
| Geovanna Badaro, MD | Yale School of Medicine, New Haven, CT, USA |
| **Connecticut HPV-IMPACT Working Group** | |
| James Meek, MPH | Connecticut Emerging Infections Program, New Haven, CT, USA |
| Monica Brackney, MS | Connecticut Emerging Infections Program, New Haven, CT, USA |
| Lynn Sosa, MD | Connecticut Department of Public Health, Hartford, CT, USA |
| Julia W. Gargano, PhD | Centers for Disease Control and Prevention, Atlanta, GA, USA. |
| Lauri E. Markowitz, MD | Centers for Disease Control and Prevention, Atlanta, GA, USA. |
| Rebecca M. Dahl, MPH | Centers for Disease Control and Prevention, Atlanta, GA, USA. |
| Juanita Onyekwuluje, MS | Centers for Disease Control and Prevention, Atlanta, GA, USA. |

| **SECTION 2. SUPPLEMENTAL FIGURES REFERENCED IN THE MAIN MANUSCRIPT** |
| --- |

## Figure S1. Flow diagram for the selection of subjects

##
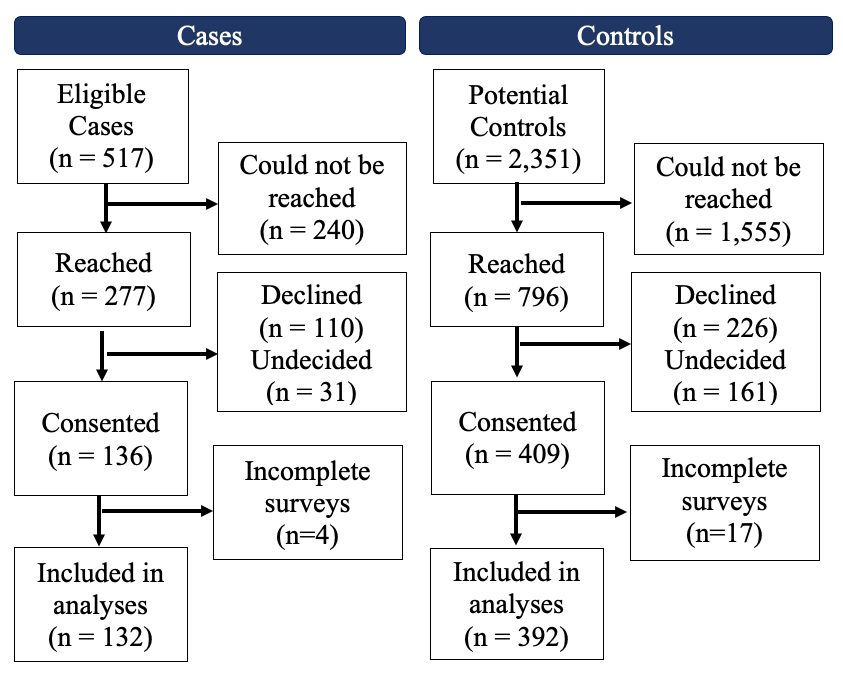


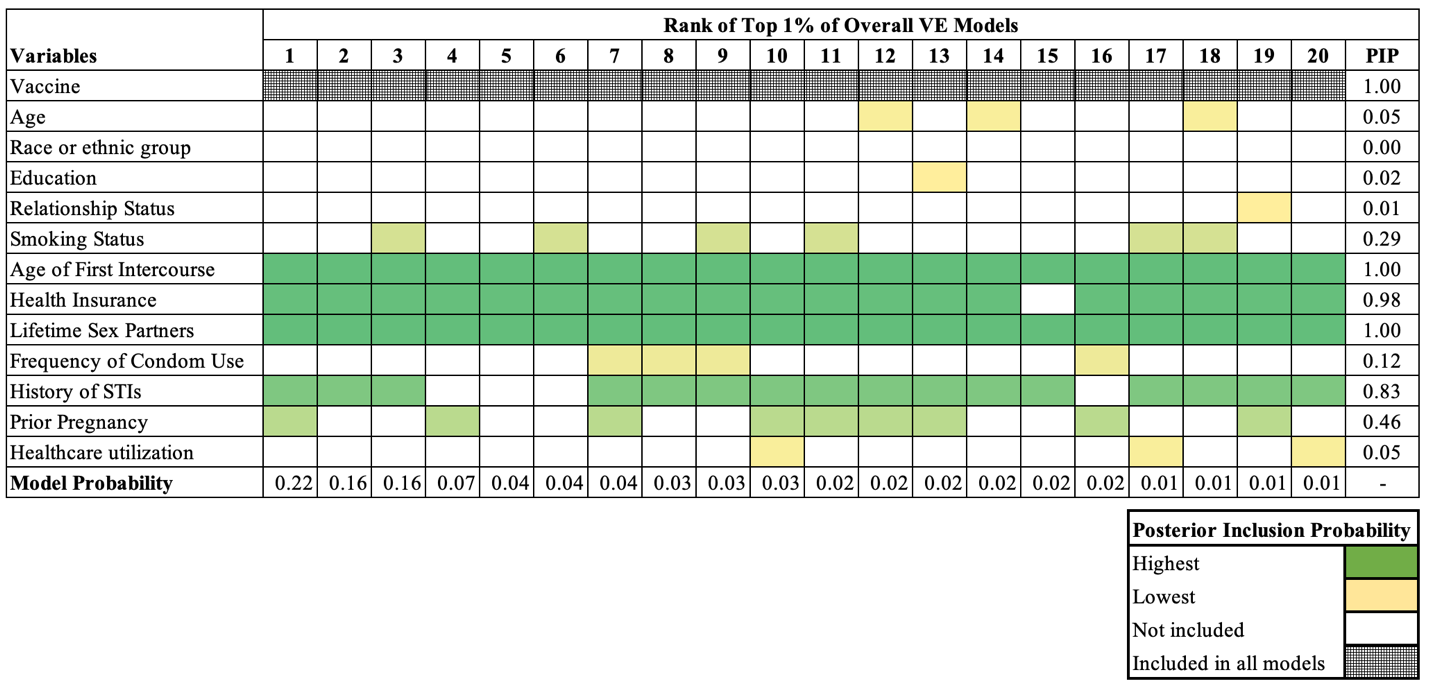


## Figure S2. Composition of top subset of candidate models used for estimating overall vaccine effectiveness

Abbreviations: VE, vaccine effectiveness; BIC, Bayesian Information Criterion; PIP, posterior inclusion probability; HPV, human papillomavirus.

This figure summarized the top 1% of all candidate models that do not adjust for age at the time of vaccination (i.e. Overall VE). Each row represents a potential confounder, and each column represents an individual model. The model with the lowest (best) BIC is on the left, and the worst model is on the right. Each square’s color represents the PIP of each potential confounder. The PIP allows for comparisons to be made about the relative importance of each potential confounder based on how it may improve the fit of the model. Unshaded squares represent confounders that were not included in a given model. The variable representing receipt of HPV vaccine (any age) was included in all models, so it is represented as gray.


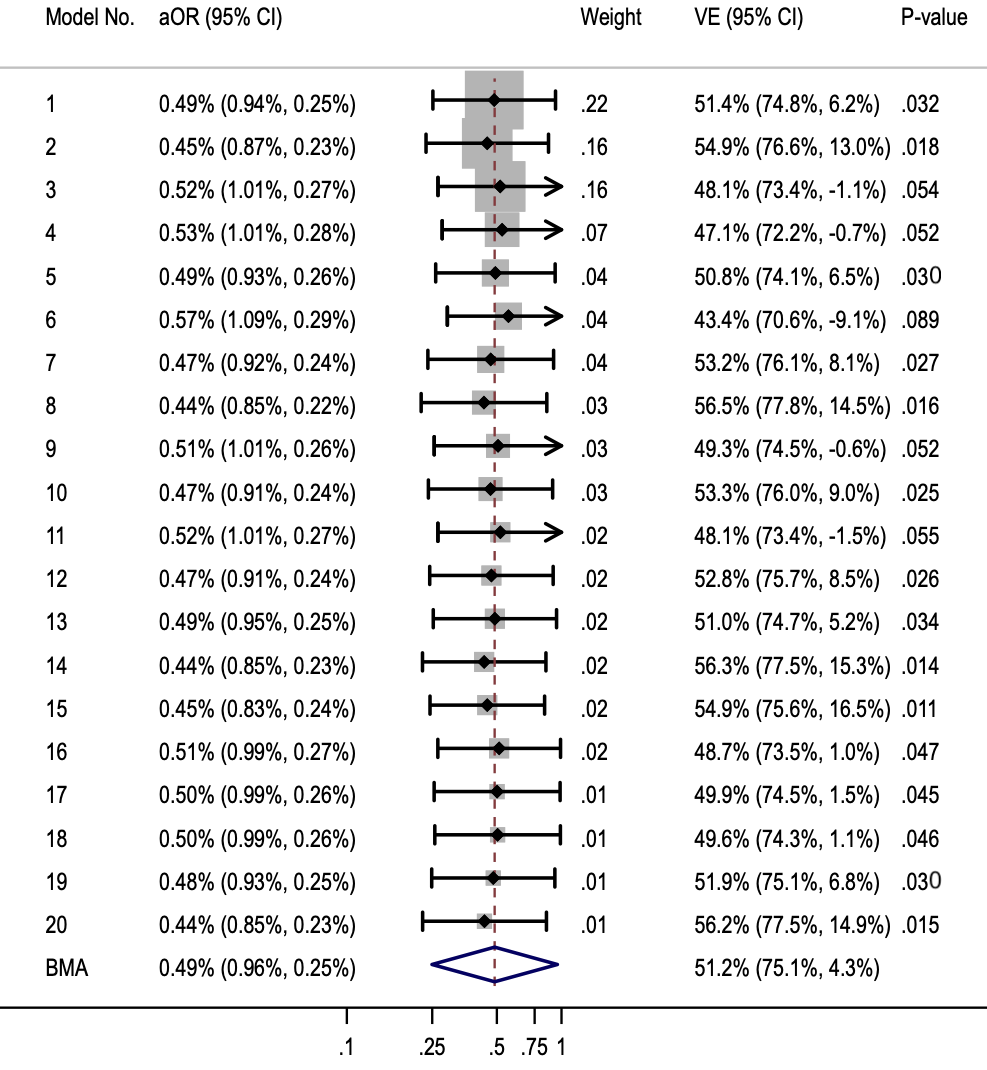


## Figure S3. Overall HPV vaccine effectiveness: model-averaged and the top subset of candidate models

Abbreviations: aOR, adjusted odds ratio; VE, vaccine effectiveness; BMA, Bayesian model averaging.

Only includes models that had a Posterior Model Probability ≥0·01.

**SECTION 3. SUPPLEMENTAL METHODS**

**Statistical analysis**

Differential participation

Descriptive analyses were first performed to evaluate if enrolled patients differed from those who were invited but declined to participate. This was achieved by computing the standardized mean difference (SMD), which is the difference in means between the two groups (enrolled versus not enrolled) in units of the pooled standard deviation.

Data on women who did not enroll were limited to what was available in the electronic medical record and surveillance system, including their age, most recent address (zip code), and race/ethnicity. Using previously described methods,^1-3^ the patient’s zip code was used as a proxy of socioeconomic status (SES) by linking it to Census data and determining whether the patient lived in a zip code that had a high, medium, or low proportion of residents with incomes below the poverty level (≥25%, 24-6% and ≤5% proportion below, respectively).

Modeling approach

We used conditional logistic regression to estimate unadjusted and adjusted odds ratios (OR) with 95% confidence intervals (CI). Vaccine effectiveness (VE) was calculated as (1 - OR) x 100%. For the primary analysis, we coded vaccination status as '1' for patients who received at least one dose of HPV vaccine two years before the focal time and '0' otherwise (including unvaccinated). We included potential confounders in our models as needed but always included vaccine status.

Multivariate models were built using the “change-in-estimate” and forward stepwise approach. To do this, we first considered each potential confounder separately by fitting numerous bivariate conditional logistic regression models. If the unadjusted OR associated with vaccination status changed by a mean of ≥10% when a given variable is included (considering all possible combinations of covariates), or if on bivariate analysis, the covariate was statistically significant using the threshold of p<0·05, the variable was treated as a potential confounder and included in final models.^4^ The final adjusted model included 6 variables: insurance, age of first intercourse, lifetime number of sex partners, smoking history, self-reported race, and history of STI.

Missingness was handled in multivariable models using listwise deletion. The rates of missing data for considered covariates ranged from 0 to 8%.

To assess variations in VE by age at the time of vaccination, we created a categorical variable coded as: '1' for patients who received their first HPV dose two years before focal time and before their 18th birthday, '2' for patients who received their first dose two years before focal time and after their 18th birthday, and '0' for all others (including the unvaccinated). We then used the beta coefficients for the different levels of the categorical vaccine variable to calculate the ORs using unvaccinated patients as the referent group.

Sample size and power

We calculated the sample sizes for different proportions of controls that might be vaccinated and for the 2:1 ratio of controls to cases using established formulas. With 132 case-control sets (2:1 ratio) and a vaccine uptake among controls of ~0·3, this study was powered (≥ 80%) to detect a minimum VE of 50% (two-tailed alpha < 0·05). Since very few participants received fewer than three doses of the vaccine, there was insufficient power (not enough discordant strata) to stratify the estimates of VE by number of doses received.

**Sensitivity analysis**

Classification of exposure or outcome

We conducted several sensitivity analyses to test the assumptions made in the primary VE analysis. To assess the impact of non-HPV-16/18 types on VE, we stratified the results based on two groups: cases with only HPV 16 or 18 and cases with another high-risk HPV type along with HPV 16/18. Additionally, we examined VE based on the severity of the cervical abnormality. To examine the effect of the 2-year buffer period used in our definition of vaccination status, we repeated the analysis using a buffer period of 6 months, 12 months, and 48 months.

Bayesian model averaging

We employed a Bayesian model averaging (BMA) framework to systematically evaluate multiple competing models and make inferences based on more than one model when estimating the VE by age at the time of vaccination. This analytical approach has been previously described in detail.^5^ Briefly, we conducted an exhaustive search for potential models by considering every possible combination of non-collinear control variables. We then used Bayesian Information Criterion (BIC) scores to approximate the posterior model probabilities for each candidate model. Next, we reduced the modeling space to include only the most realistic models by dropping models that provided little to no explanatory power (i.e., those with a cumulative probability of <0·01 relative to the best model). BIC-weights were then computed for the newly defined set of models (i.e., the top set), and a BMA-VE (with 95% credibility intervals) was calculated by exponentiating the weighted average of the vaccine coefficient and its standard error. This approach not only offers an assessment of the robustness of the VE point estimate but also enables the estimation of the effect confounding factors have on VE. Estimating confounder influence involves evaluating the change in the vaccine coefficient 𝛽1 when a given confounder was incorporated into the model.

The components of each model, their BIC-derived weights (i.e., their model probability), and the posterior inclusion probability of each potential confounder are summarized in **Figure S1**. This heatmap illustrates that the significance of a variable does not always correspond with the variable's impact on the VE point estimate. Among the 12 potential confounders that were considered, age of first intercourse, health insurance, and lifetime number of sex partners all had a posterior inclusion probability (PIP) of 1·00, which suggests that they have considerable explanatory power and should be included in models assessing VE using this dataset. However, race/ethnicity and smoking history were the variables that exhibited the most influence on the point estimate (ΔOR= -10% and 9%, respectively). The point estimates and confidence intervals for the top subset and the model-averaged VE are shown in **Figure S2**. The adjusted OR was consistent across the top subset of models, with 95% confidence intervals crossing the null in only 5/20 models. The consensus overall VE estimate using BMA was 51% (95% CI: 4-75%).

**HPV Detection and Typing**

HPV Vaccine Impact Monitoring Project Across CT (HPV-IMPACT) surveillance system was the source of case ascertainment for this vaccine effectiveness study. HPV-IMPACT was established in 2008 as a collaboration between the Connecticut Department of Public Health, the Yale School of Public Health, and the Centers for Disease Control and Prevention (CDC). The project is a CDC-funded initiative that conducts population-based surveillance of HPV and cervical precancers. In Connecticut, surveillance involves gathering clinical data and biospecimens from all pathology laboratories that serve residents of the state. Only a subset of the patients in the HPV-IMPACT surveillance system were eligible to participate in this case-control study. Specifically, eligible women were those who: 1) were born on or after 1981, 2) were residents of New Haven County, 3) received care within the Yale New Haven Health System (YNHHS), and 4) were diagnosed with HGCL that tested positive for HPV 16 or HPV 18. As part of this project, tissue blocks that were initially used to diagnose the HGCL as part of routine clinical care are collected from pathology laboratories and transported to the CDC for histopathologic evaluation and HPV testing using standard procedures. DNA extraction from formalin-fixed paraffin embedded tissues sections was performed at CDC as previously described.^6^ A pathologist at the CDC examined all cervical tissue samples to confirm the presence of diagnostic material. Only biopsies with adequate diagnostic material underwent HPV typing. In cases where cervical biopsy samples sent to the CDC were not representative of the lesion or if CDC results were not available, HPV results on the trigger pap performed at Yale Pathology as part of routine clinical care were used. Yale Pathology utilized a multiplex real-time polymerase chain reaction (PCR) system (Cobas 4800, Roche Diagnostics, Pleasanton, CA) which individually detects HPV types 16 and 18, and detects 12 other high-risk types as a pooled result (31, 33, 35, 39, 45, 51, 52, 56, 58, 59, 66, and 68).

# SECTION 4. Supplement References

1. Gross CP, Filardo G, Mayne ST, Krumholz HM. The impact of socioeconomic status and race on trial participation for older women with breast cancer. *Cancer*. Feb 1 2005;103(3):483-91. doi:10.1002/cncr.20792

2. Link-Gelles R, Westreich D, Aiello AE, et al. Bias with respect to socioeconomic status: A closer look at zip code matching in a pneumococcal vaccine effectiveness study. *SSM Popul Health*. Dec 2016;2:587-594. doi:10.1016/j.ssmph.2016.08.005

3. Oliveira CR, Avni-Singer L, Badaro G, et al. Feasibility and Accuracy of a Computer-Assisted Self-Interviewing Instrument to Ascertain Prior Immunization With Human Papillomavirus Vaccine by Self-Report: Cross-Sectional Analysis. *JMIR Med Inform*. Jan 22 2020;8(1):e16487. doi:10.2196/16487

4. Greenland S. Modeling and variable selection in epidemiologic analysis. *Am J Public Health*. Mar 1989;79(3):340-9. doi:10.2105/ajph.79.3.340

5. Oliveira CR, Shapiro ED, Weinberger DM. Bayesian Model Averaging to Account for Model Uncertainty in Estimates of a Vaccine's Effectiveness. *Clin Epidemiol*. 2022;14:1167-1175. doi:10.2147/CLEP.S378039

6. Hariri S, Unger ER, Powell SE, et al. Human papillomavirus genotypes in high-grade cervical lesions in the United States. *J Infect Dis*. Dec 15 2012;206(12):1878-86. doi:10.1093/infdis/jis627
